# COVID-19 ERA EFFECTS ON OLDER ADULTS’ COGNITIVE COMPLAINTS, DEPRESSIVE SYMPTOMS, AND STRESSFUL EVENTS

**DOI:** 10.1093/geroni/igac059.1689

**Published:** 2022-12-20

**Authors:** Suzanne Segerstrom, Paris Crosby, Dakota Witzel, Maria Kurth, Soyoung Choun, Carolyn Aldwin

**Affiliations:** University of Kentucky, Lexington, Kentucky, United States; University of Kentucky, Lexington, Kentucky, United States; Penn State University, State College, Pennsylvania, United States; Oregon State University, Corvallis, Oregon, United States; Oregon State University, Corvallis, Oregon, United States; Oregon State University, Corvallis, Oregon, United States

## Abstract

Older adults have adjusted better to the COVID-19 pandemic in terms of their psychological well-being than younger adults. We investigated individual differences in vulnerability within older adulthood as pandemic severity changed, providing a more refined prediction of older adults’ adjustment to COVID-19. Participants from this longitudinal study were included if they had at least one semiannual assessment before and one during the COVID-19 era (N = 111, 65% women, age range = 62-96 at onset of COVID-19 era in the US). There were 1,098 pre-COVID-19 assessments (M=9.9, 1/5/2018-1/22/2020) and 265 post-COVID-19 (M=2.4, 1/23/2020-10/31/2021). At each assessment, participants reported on six cognitive complaints (MOS), five depressive symptoms (Geriatric Depression Scale), and six domains of undesirability-weighted stressful life events (Louisville Older Persons Event Scale). Daily national, state, and regional COVID-19 case and death rates were obtained from the Centers for Disease Control and summed for the week preceding each assessment. In multilevel ZIP models, the COVID-19 era significantly increased depressive symptoms (0.68 to 1.18, p < .0001) and stressful events (30.9 to 48.5, p < .0001), but did not significantly affect severity of cognitive complaints. Older age was associated with greater impact of COVID-19 on depressive symptoms and stressful events; women reported more stressful events when pandemic severity was high, but men reported more stressful events when pandemic severity was low. Although older adults in general have adjusted better to the pandemic than younger adults, the old-old had greater vulnerability to this unavoidable event than the young-old.

